# Targeting Prion-like Cis Phosphorylated Tau Pathology in Neurodegenerative Diseases

**DOI:** 10.4172/2161-0460.1000443

**Published:** 2018-06-29

**Authors:** Onder Albayram, Peter Angeli, Elizabeth Bernstein, Sean Baxley, Ziang Gao, Kun Ping Lu, Xiao Zhen Zhou

**Affiliations:** 1 Division of Translational Therapeutics, Department of Medicine, Beth Israel Deaconess Medical Center, Harvard Medical School, 330 Brookline Avenue, CLS 0408, Boston, MA 02215, USA; 2 Cancer Research Institute, Beth Israel Deaconess Medical Center, Harvard Medical School, 330 Brookline Avenue, CLS 0408, Boston, MA 02215, USA; 3 Broad Institute of Harvard University and Massachusetts Institute of Technology, Cambridge, MA 02142, USA; 4 Fujian Key Laboratory for Translational Research in Cancer and Neurodegenerative Diseases, Institute for Translational Medicine Fujian Medical University, Fuzhou, Fujian, China

**Keywords:** Traumatic brain injury, Alzheimer’s disease, Prion, Tau, Pin1

## Abstract

Tau is a microtubule-associated protein heavily implicated in neurodegenerative diseases collectively known as tauopathies, including Alzheimer’s disease and chronic traumatic encephalopathy. Phosphorylation of tau at Thr231 allows for the isomerization of phosphorylated tau (p-tau) into distinct cis and trans conformations. Cis, but not trans, p-tau is detectable not only in Alzheimer’s disease and chronic traumatic encephalopathy, but also right after traumatic brain injury depending on injury severity and frequency both in humans and animal models. Cis p-tau is not only neurotoxic but also spreads from a neuron to another in a prion-like fashion, functioning as a primary driver of neurodegeneration, which can be effectively neutralized by cis p-tau antibody. This represents an exciting new opportunity for understanding disease development and developing early biomarkers and effective therapies of tauopathies.

## Overview of Prions and Neurodegenerative Disease

The accumulation of protein aggregates is a common feature of many neurodegenerative diseases including Alzheimer’s disease (AD), Parkinson’s disease (PD) and frontotemporal dementia (FTD) [1], Each type of aggregate has one type of protein as its major component, with amyloid-β, hyperphosphorylated tau and α-synuclein being the most commonly observed. These proteins undergo a transformation from a soluble monomer to an insoluble, aggregated state through a number of intermediates [2]. Researchers have speculated that the protein deposits found in these neurodegenerative diseases may develop and spread throughout the brain in a manner analogous to that of aggregation of the prion protein (PrP) in transmissible spongiform encephalopathies (TSEs), such as Creutzfeldt-Jakob disease (CJD) [1,3]. Many recent studies in rodents [4–7], as well as in humans [8,9], support the notion that AD pathology propagates in a prion-like fashion [2]. Elowever, there is no evidence suggesting that non-TSE neurodegenerative diseases, including AD, can be transferred between individuals in any case other than direct injection of diseased brain extracts, hence the use of “prion-like.”

Experiments involving intracerebral or intraperitoneal injections of AD brain extracts into susceptible mice have been shown to induce cerebral amyloidosis and associated pathology in a donor- and host-dependent manner, indicating prion-like features of amyloid-β (Aβ) [10]. The misfolded amyloid protein within diseased brain extracts appears to be able to seed the misfolding of Aβ in the new host, driving the accumulation of Aβ aggregates, a defining feature of AD, in several brain regions. The prion-like nature of Aβ pathology in humans was further verified through the autopsy of patients between the ages of 36 and 50 who had died of Creutzfeldt-Jakob disease (CJD) contracted as a result of treatment, typically in childhood, with human cadaveric pituitary-derived growth hormone contaminated with prions [11]. Several of these patients, none of whom had high-risk alleles for early-onset Alzheimer’s, showed substantial amyloid-beta pathology, which is extremely uncommon for this age group. Because the pituitary gland shows amyloid pathology in a subset of Aβ+ patients, this discovery strongly suggests that the growth hormone was contaminated with pathogenic amyloid-beta and that the amyloid-beta was then transmitted through the same mechanism as the prions which caused the patients’ CJD [8].

For 25 years most research has focused on the amyloid hypothesis of AD pathogenesis and progression, which states that Aβ is the primary driver and neurotoxic element of AD. Recent roadblocks to the progression of Aβ-targeted therapies, along with the new concept of prion-like propagation of intracellular abnormal proteins, have brought tau into the spotlight as a potential therapeutic target [12–20]. Moreover, tau aggregation also plays a role in many other neurodegenerative disorders, collectively known astauopathies. These include Pick’s disease (PiD), progressive supranuclear palsy (PSP), dementia with Eewy bodies (DEB), and corticobasal degeneration (CBD) [21,22]. Insight into the propagation and toxicity mechanisms of abnormally folded tau protein has the potential to offer promising new therapeutic targets for a number of disorders.

## Structure and Toxicity of Pathological Tau

Native tau has a relatively loose, unstructured protein with little α-helix and β-sheet structure. In the adult human brain, tau protein appears as six isoforms, all derived from a single gene by alternative splicing. Three of these isoforms contain three repeats (3R-τ) of a sequence thought to be involved in binding to microtubules; the other three isoforms contain an additional fourth repeat of the region, coded by exon 10 (4R-τ). The repeat region of tau is positioned between two basic, proline rich regions. Many of these prolines are preceded by a serine or threonine, allowing for phosphorylation. Dimerization due to disulfide cross-linking has been proposed to be a first step in the formation of NFTs, and only occurs when lysines in the microtubule binding repeat regions are phosphorylated [23]. This in turn disrupts tau’s function on microtubules and alters its protein stability, eventually leading to aggregation and tangle formation [24].

Neurofibrillary tangles are a neuropathological hallmark of tauopathies AD and other tauopathies and were previously believed to be the toxic species. However, recent studies have demonstrated that cell death occurs before tangle formation, meaning some earlier intermediate must be the source of tau toxicity [25]. The culmination of many years of increasing research into the toxicity of tau aggregation in neurodegenerative disease has led to the proposal that soluble, oligomeric forms of hyperphosphorylated tau (p-tau) are likely the most toxic entities in disease [21]. These p-tau oligomers are able to enter and exit cells *in vitro*, and are believed to be a major species responsible for propagation, although the exact mechanism is still unknown [25–27]. Evidence suggests that these multimeric tau oligomers may act as templates for the misfolding of native tau, thereby seeding the spread of the toxic forms of the protein and initiating disease progression in a manner analogous to that of prions [28,29]. Additionally, these oligomers have repeatedly been found to induce cell death in numerous tauopathies and can propagate through the brain causing synaptic and mitochondrial dysfunction associated with memory deficits [30,31].

**Figure 1:**
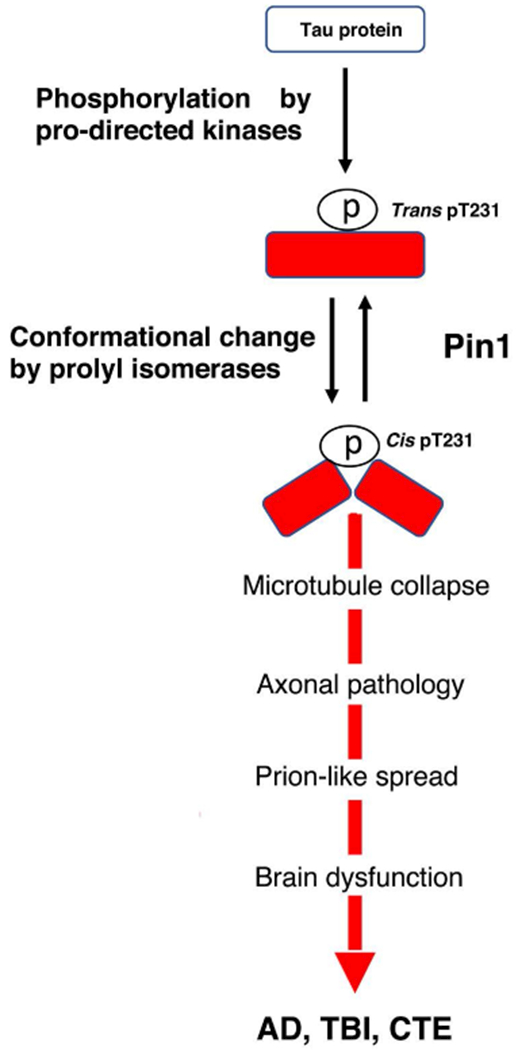
Upon phosphorylation of tau on the Thr231-Pro motif, Pin1 converts cis to trans of p-tau at a much higher frequency, although it can catalyze both directions. When Pin 1 becomes down-regulated or when cis p-tau is produced in abnormally high quantities, as we see in the case in TBI and AD, the cis conformation begins to accumulate in the brain. Unlike trans p-tau, which can bind and promote microtubule assembly, is susceptible to protein dephosphorylation and degradation and resistant to protein aggregation, and does not cause neurodegeneration, cis p-tau cannot bind and promote microtubule assembly, is resistant to protein dephosphorylation and degradation, prone to protein aggregation, and cause and spread neurodegeneration.

Physiologic tau is not capable of aggregating or causing the pathology observed in tauopathies. In order to find more effective therapeutic targets and treatments, any uncharacterized prion-like variants of tau, and other proteins associated with neurodegenerative diseases, must be identified. Our studies have led us to believe that cis p-tau is the soluble tau variant responsible for toxicity [32]. After protein phosphorylation on specific serine or threonine residues preceding a proline (pSer/Thr-Pro), the function of certain phosphorylated proteins is further regulated by a cis-trans conformational change around the Ser/Thr residue [33,34]. Cis, but not trans, pThr231-tau appears early in mild cognitive impairment (MCI) neurons and further accumulates only in degenerating neurons as AD progresses, localizing to dystrophic neurites, which are known to correlate well with memory loss [35]. Unlike trans p-tau, the cis conformation cannot promote microtubule assembly, is more resistant to dephosphorylation and degradation, and is more prone to aggregation [35]. Pin1, a peptidyl-prolyl cis-trans isomerase, binds to phosphorylated tau primarily at the Thr231 residue, catalyzing the conversion from cis to trans p-tau at that site [32–43] (Figure 1). When Pin1 becomes down regulated, the cis conformation of tau can accumulate [41].

## Alzheimer’s Disease

The work from our lab and others has uncovered the extensive contribution ofPin1 to the development of AD pathology. Pin1 promoter polymorphisms resulting in decreased Pin1 levels are associated with an increased risk for late-onset AD [44]. In contrast, Pin1 SNPs resulting in reduced Pin1 inhibition have been associated with a delayed onset of AD [40]. In a normal human brain, Pin1 expression was relatively low in regions of the hippocampus that are susceptible to NFT-related neurodegeneration in AD (CA1 and subiculum), while Pin1 expression was higher in regions that are generally spared (CA4, CA3, CA2, presubiculum) [42]. In the brains of human AD patients, the majority of pyramidal neurons (96%) with relatively high Pin1 expression lacked tau tangles, and most pyramidal neurons (71%) with relatively low Pin1 expression had tangles [42]. Furthermore, Pin1 co-localizes and co-purifies with NFTs, and can directly restore the ability of tau to bind microtubules and promote microtubule activity [36,45]. Finally, levels of p-Thr231 tau correlate with the progression of AD, and Pin1 is strongly correlated with dephosphorylation of tau at Thr231 [43,46].

**Figure 2:**
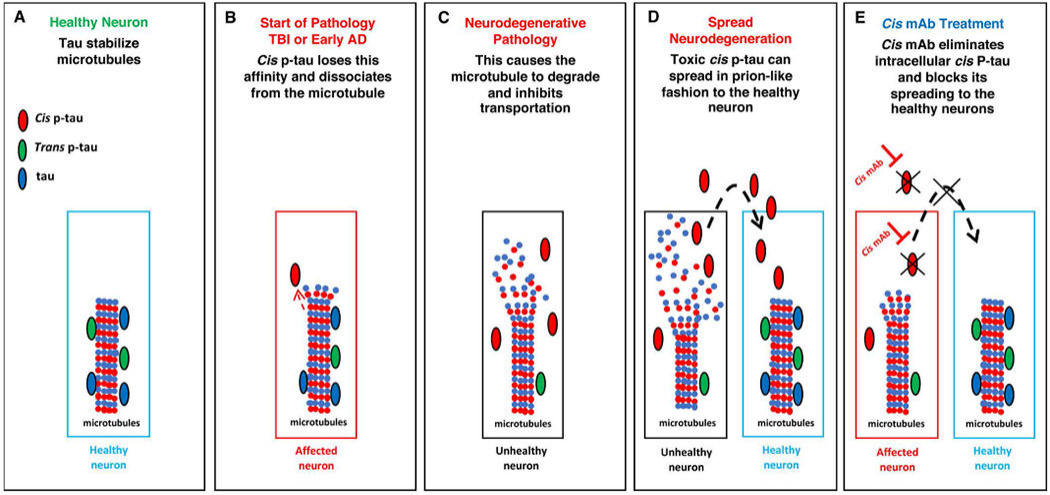
(A) Healthy tau has a high affinity for microtubules, promoting their assembly and assisting in the transportation of various proteins and nutrients along the axon. (B) Cis p-tau loses this affinity and dissociates from the microtubule. (C) The tau which dissociates from the microtubule also develops a higher affinity for itself and begins to aggregate, and actually seeds itself in a manner similar to the prion protein. This essentially means that toxic tau can convert other healthy tau in the brain into the toxic cis conformation, causing a systematic and predictable spread of tau pathology. This cis P-tau may be the missing step in the process of neurodegeneration. (D) The isomerization causes healthy, physiological trans tau to become a toxic cis form, which is capable of causing and spread neurodegeneration and eventual tau tangles in tauopathies. (E) The ability of cis p-tau to cause and spread neurodegeneration can be effectively neutralized by cis p-tau antibody, which targets intracellular cis P-tau for proteasome-mediated degradation and preventing extracellular cis P-tau from spreading to other neurons.

Behind all the effects of Pin1 is a conformational change between cis and trans. In 2012, by developing the first antibodies able to distinguishing the cis from trans pThr-Pro motif of tau, our group discovered that Pin1 specifically converts cis hyperphosphorylated tau, a conformation that can no longer promote microtubule assembly, to the physiologic trans [38]. Cis p-tau is more stable, less susceptible to dephosphorylation, and more prone to aggregation than trans p-tau [38]. The same study detected little to no cis or trans in healthy brains, but a dramatic increase in cis p-tau in the brains of AD patients [38]. Additionally, cis, but not trans, p-tau was strongly present in the brains of mild cognitive impairment (MCI) patients [38]. Both cis and trans p-tau were found in the cell bodies of AD neurons, but only cis p-tau was present in neuritis [38]. Besides, all cis p-tau positive cells in the hippocampus were also positive for a marker of neurofibrillary neurodegeneration, while the majority of neurons trans positive cells were negative for the marker [38]. Together these results strongly suggest that cis p-tau is an early, strong driver for AD pathology and neurodegeneration (Figure 2). In turn, the brain is protected from both cis p-tau and harmful amyloid processing by the activity of Pin1.

## Traumatic Brain Injury and Chronic Traumatic Encephalopathy

Traumatic brain injury (TBI) and chronic traumatic encephalopathy (CTE) are closely related tauopathies that have significant potential for studying the toxicity and spread of tau in controlled conditions. Repetitive mild TBI (rmTBI), or single moderate/severe TBI (ssTBI), may cause acute and potentially long-lasting neurological dysfunction, including the development of CTE [47–49]. Additionally, TBI is an established environmental risk factor for AD [50].

The predictable and systematic nature of the progression of this tau pathology has made it possible identify 4 distinct stages of CTE. In stage I CTE, p-tau pathology is found in discrete foci in the cerebral cortex, most commonly in the superior or lateral frontal cortices, typically around small vessels at the depths of sulci. In stage II CTE, there are multiple foci of p-tau at the depths of the cerebral sulci and there is localized spread of neurofibrillary pathology from these epicenters to the superficial layers of adjacent cortex. In stage III CTE, p-tau pathology is widespread with the frontal, insular, temporal and parietal cortices showing widespread neurofibrillary degeneration. The greatest severity of this degeneration is located in the frontal and temporal lobes, concentrated at the depths of the sulci. Also in stage III CTE, the amygdala, hippocampus and entorhinal cortex show substantial neurofibrillary pathology that is found in earlier CTE stages. In stage IV CTE, there is widespread severe p-tau pathology affecting most regions of the cerebral cortex and the medial temporal lobe, sparing calcarine cortex in all but the most severe cases [51].

This systematic progression through connected brain regions is considered prion-like and quite similar to the spread of tau in Alzheimer’s. However, different specific regions of the brain are affected, and in a different order, in the two diseases. This difference could be caused by different strains of cis p-tau acting by the same mechanism, and/or by different sites of initial pathology (the entorhinal cortex in AD, and the isocortex in TBI/CTE).

By generating a monoclonal antibody (mAh) pair capable of distinguishing between cis and trans isoforms of p-tau (cis p-tau and trans p-tau, respectively), cis p-tau was identified as a precursor of tau pathology and an early driver of neurodegeneration in traumatic brain injury (TBI) and chronic traumatic encephalopathy (CTE) [52]. Notably, cis p-tau appears within hours after closed head injury and long before other known pathogenic p-tau conformations including oligomers, pre-fibrillary tangles and NFTs [52]. Neurons labeled with antibodies that recognize phospho-Thr231 and phospho-Ser262 display significantly decreased normal physiological interactions between tau and microtubules upon tau phosphorylation [53–55], which can lead to the destabilization of microtubules and eventually apoptosis (Figure 2). Importantly, other groups have independently confirmed that the pT231-tau level in human blood is a novel biomarker for acute and chronic traumatic brain injury that is correlated with poor outcome in patients [56,57].

## Therapy

Studies have revealed the potential of antibody treatments in neutralizing toxic cis p-tau, thereby halting, or at least significantly delaying, neurodegeneration. This therapy has so far proved successful in mouse models of TBI [32,52,58]. Periodic treatment with a cis p-tau monoclonal antibody treatment over 4 months not only eliminated spreading of cis p-tau, axonal pathology and astrogliosis into the hippocampus without affecting physiologic trails p-tau, but also prevented tau oligomerization, tangle formation, and APP accumulation (Figure 2) [52,58]. Shorter courses of treatment (between 5 and 10 days) with delayed administration also proved effective at eliminating cis p-tau induction [58]. A humanized version of the cis p-tau antibody could prove extremely useful in treating the wide range of neurodegenerative diseases associated with toxic tau.

Multiple prion strains can exist in one patient, and the various strains often compete with each other [59,60]. This competition was recently explored as a potential therapy, where the introduction of a non- or less-toxic prion strain protected against the propagation of the toxic strain [61]. The application of this finding to the prion-like propagation of tau through overexpression of non-toxic trans-tau could be promising but requires further investigation.

## Conclusion

For many years NFTs were the main subject of study in research done on tau toxicity in neurodegenerative diseases. Recently evidence has pointed to an intermediate in the aggregation process, such as soluble cis p-tau, as a more likely candidate for the toxic species in common tauopathies. As more research has been done on tau aggregation intermediates, the prion-like nature of tau has been made apparent. Toxic tau is able to seed and spread itself through connected regions of the brain, displaying systematic and predictable patterns of spread in various tauopathies, similar to the patterns of spread seen in TSEs caused by misfolded prion protein. The difference between the tau pathology in the various tauopathies is thought to be caused by different strains or conformations of the toxic, misfolded tau. Our studies have indicated that inhibition of Pin 1-mediated isomerization of cis p-tau to trans p-tau may induce tau toxicity. The use of mouse monoclonal antibodies that can distinguish trans p-tau from cis p-tau has shown promise as a potential method for both characterization and treatment of tau pathology. Once more is known about the prion-like nature of tau, and exactly how similar tau spread is to that of PrP, the strain competition model of therapy, which is currently being tested in TSEs, could potentially be applied to tauopathies. Understanding the prion-like mechanisms of the tau protein will be an important step in discovering new and more effective treatments for many common neurodegenerative diseases.
